# A test-retest reliability study of assessing small cutaneous fibers by measuring current perception threshold with pin electrodes

**DOI:** 10.1371/journal.pone.0242490

**Published:** 2020-11-17

**Authors:** Weiwei Xia, Han Fu, Haiying Liu, Fanqi Meng, Kaifeng Wang

**Affiliations:** 1 Department of Spinal Surgery, Peking University People’s Hospital, Beijing, China; 2 Department of Respiratory Medicine, Chinese People’s Liberation Army (PLA) General Hospital, Beijing, China; Universita degli Studi di Roma La Sapienza Facolta di Medicina e Psicologia, ITALY

## Abstract

**Background:**

The quantitative measurement of current perception threshold (CPT) has been used as a method to assess the function of nerve fibers in neuropathy diseases. The aim of this study was to assess the test-retest reliability measuring CPT using the circular pin electrodes for assessing the function of cutaneous thin nerve fibers.

**Methods:**

CPT measurement was repeated on two separate days with at least one-week interval in 55 volunteers. Superficial blood flow (SBF) and skin temperature (ST) were measured on the skin in an around area concentric to the circular pin electrodes after the process of finding CPTs. The coefficient of variation (CV) and intra-class correlation coefficient (ICC) were calculated. The correlation between each two of CPT, SBF increment and ST increment was analyzed.

**Results:**

No significant differences were found for CPT, SBF and ST between two sessions. SBF was found to be significantly increased after the process of finding CPT. CPT values of males were found to be higher than females. SBF increment was found to be positively correlated with ST increment. The ICC values for CPT, SBF and ST were 0.595, 0.852 and 0.728, respectively. The CV values for CPT, SBF and ST were 25.53%, 12.59% and 1.94%, respectively.

**Conclusions:**

The reliability of CPT measurement using circular pin electrodes is fair, and need consistence of measurements in longitudinal studies.

## Introduction

The quantitative measurement of current perception threshold (CPT) has been used as a method to assess the function of nerve fibers in neuropathy diseases [1, 2]. CPT refers to the minimum current stimulation intensity applied by the skin that induces the subject to produce sensations. Compared to traditional sensory measurements by physical stimulations, such as light touch using brush, vibration using tuning fork and pinprick stimulation, CPT measurement as an electro-diagnostic testing has been shown to be more sensitive for detecting sensory deficit [2, 3]. Many diseases at early stage of lesions can selectively damage a certain type of sensory nerve fiber (A-β/A-δ/C-type nerve fibers), e.g., diabetic neuropathy generally appears first in the small unmyelinated fibers and it can predate many months before any evidence of damage of the larger fibers [4]. CPT measurement was used to selectively test the functional integrity of different fiber types by different stimulation paradigms, such as the Neurometer^®^ device (2000 Hz for A-β fibers, 250 Hz for A-δ fibers, 5 Hz for C-fibers) [1, 2]. CPT test can be used to evaluate the function of the neural transmission by testing different nerve fibers. Therefore, small fiber neuropathy may be reversed or significantly improved with appropriate intervention by early diagnosis with CPT examination. In addition, CPT test can be used to observe the development of neuropathy [5]. This can help to take timely intervention and improve the prognosis.

Previous studies showed that compared to surface patch electrode, the circular pin electrode can preferably active A-δ nerve fibers [6, 7]. The Neurometer^®^ electrodes also have a relatively larger surface compared to the pin electrodes. It has a degree of neuroselectivity but also have a large variability [8]. The sensation of electrical stimulation by pin electrodes is a needling sensation, whereas the surface patch electrode causes a pressing sensation [6, 9, 10]. Furthermore, low intensity of intra-epidermal electrical stimulation has been verified to be able to activate epidermal A-δ fibers [11]. The mechanism of preferential activation of small nerve fibers is that high current density locates in the epidermis and most small nerve fibers (e.g., A-δ/C-fibers) terminate there, whereas non-nociceptive large nerve fibers (A-β fibers) terminate deeper in the dermis [6]. The high epidermal current density achieved through the pin electrodes by low intensity electrical stimulation is enough to overcome the higher activation threshold of the nociceptors [6]. Therefore, the pin electrodes could be used to assess the function of cutaneous small fibers.

In addition, the non-painful electrical stimulation was reported to be able to increase superficial blood flow (SBF) and skin temperature (ST) which stand for the neurogenic inflammation responses [12]. Neurogenic inflammation is correlated to the activation of peptidergic afferents (e.g., A-δ/C-fibers), which can release neuropeptides such as calcitonin gene-related peptide (CGRP) or substance P (SP) that increase superficial blood flow [13]. Therefore, SBF and ST could be used as a manifestation of neurogenic inflammation after electrical stimulation through pin electrodes as peripheral afferents can be activated leading to the release of neurogenic transmitters [14–17]. In addition, it has been reported that gender could affect CPT values [18].

However, a test-retest study needs to be conducted to assess the reliability of measuring CPT using the circular pin electrodes. The traditional reliability tests include assessments of relative and absolute reliability [19]. Relative reliability refers to the degree in which individuals maintain their position over repeated measurements measured by intra-class correlation coefficient (ICC); absolute reliability refers to the degree in which repeated measurements vary for individuals measured by coefficient of variation (CV) and Bland-Altman analysis [19, 20].

The aims of the present study were to:1) assess the reliability of CPT measured by pin electrodes; 2) analyze the relationships between CPT and superficial blood flow (SBF) and skin temperature (ST); 3) analyze the gender effect on CPT values. This will help to guide the measurements of CPT using circular pin electrodes in human studies.

## Materials and methods

### Ethical approval

The ethical approval was obtained from the Medical Ethics Committee of Peking University People’s Hospital (2019PHB151-01). All subjects gave their written informed consent prior to their inclusion in the study. The study was performed according to the Declaration of Helsinki. This study was not registered in a database.

### Study population

The experiments were performed on 55 subjects (21 females and 34 males:20-47years; mean age 26.75 years) after obtaining the ethical approval. All subjects participated in a familiarization session and two experimental sessions with the same procedures and measurements. Each session lasted for about half an hour. This study was conducted from October to December in 2019. The present study included a large number of participants, which adds weight to the data. Exclusion criteria were prior or current skin disease, neurological disease, any history of chronic pain as well as drug abuse or suffering from ongoing pain.

### Experiment protocol

**Fig 1:**
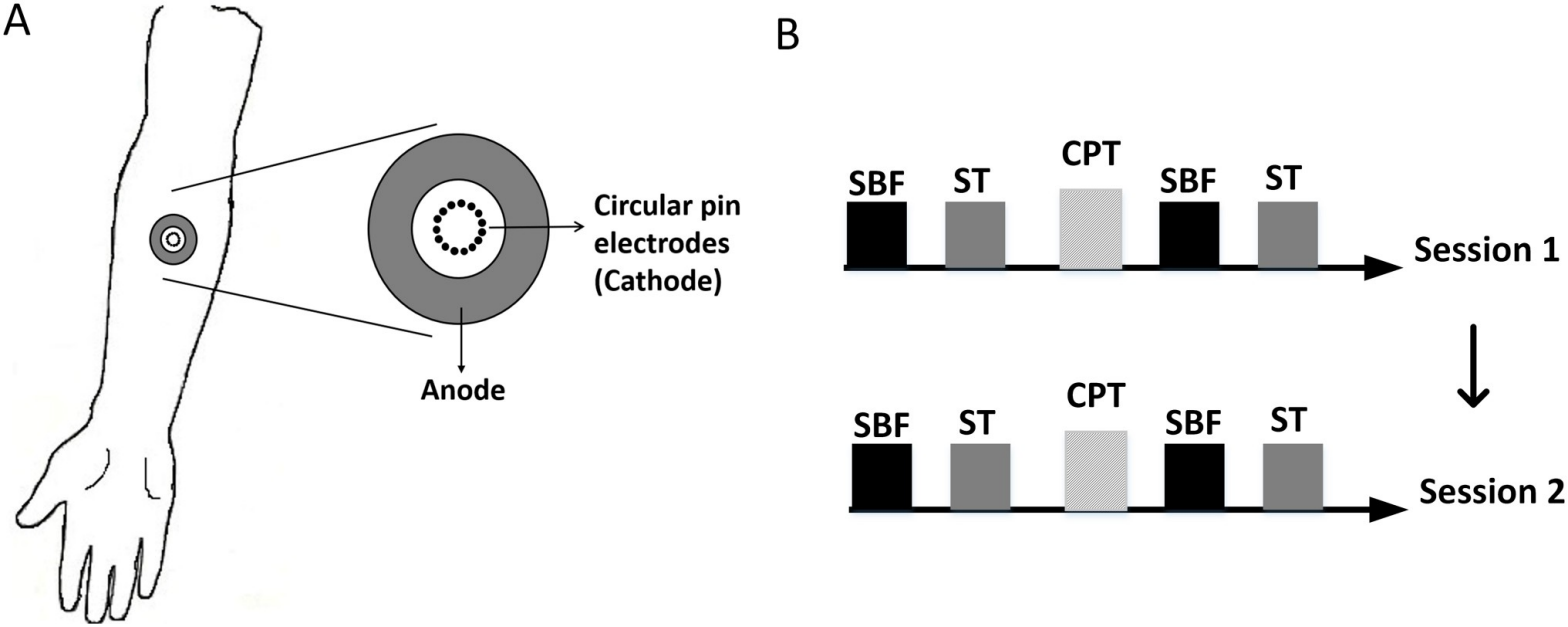
Experimental setup. A. The circular pin electrodes was applied at the volar forearm 7 cm distal to the cubital fossa. B. Superficial blood flow (SBF) and skin temperature (ST) were assessed before and after current perception threshold (CPT) measurements which were repeated on two different days separated by at least one week for the individual subject.

Three sessions were arranged for each subject. The first familiarization session was aimed to make the subjects familiar with the electrical stimulus modalities and gaining experience on how to use the equipment to help finding CPT in time and accurately. The experimental procedures included CPT measurements, superficial blood flow (SBF) and skin temperature (ST) assessments. The experiment was repeated on two different days separated by at least one week for the individual subject to eliminate the habituation and tiredness of the subjects. (Fig 1B). The same researcher performed all experiments to rule out the inter-rater variation with a single-blinded study design.

### CPT measurements

Transcutaneous electrical stimulation from a constant current stimulator (DS5; Digitimer Ltd; Welwyn Garden City, UK) was applied in the right forearm 7 cm distal to the cubital fossa. The stimulations were applied with an epicutaneous pin electrode consisting of a circular array (diameter: 10 mm) of fifteen cathodal electrodes each with diameter of 0.2 mm, protruding 1 mm from the base, and a large circular stainless steel plate served as an anode with an inner diameter of 20 mm and an outer diameter of 40 mm placed concentrically around the cathodes (Made in Aalborg University, Denmark) (Fig 1A) [7, 9]. The electrode was fixed by a tape after placed on the forearm. CPT was determined using the method of limits [21, 22]. The intensity of stimulation pulse started from 20 μA. The electrical stimulation frequency was 1 Hz and the pulse duration was 1 ms in rectangular wave form. It has been shown that the selective activation of small fibers (e.g., A-δ fibers) is increased with long pulse durations (i.e., longer than 0.4 ms) [7]. The current was delivered through the 15 pin electrodes at the same time. Then the electrical stimulation intensities increased with at step sizes of 3% of the previous stimulation intensity. Subjects pressed a response button to stop electrical stimulation as soon as they felt the sensation of electrical stimulation, i.e., a mild needling sensation with no pain. The first CPT value was recorded. Then the electrical stimulation intensity increased 10%, after which, the intensity decreased at step sizes of 3% of the previous stimulation intensity. Subjects pressed the response button to stop electrical stimulation as soon as they could not feel the electrical stimulation. The second CPT value was recorded. Then the simulation intensity reduced 10%, and the intensity increased at step sizes of 3% again. This process was repeated three times and six CPT values were obtained. The average of six CPT values was considered to be the final CPT value.

### Superficial blood flow

Full-Field Laser Perfusion Imager (MoorFLPI; Moor Instruments Ltd, Axminister, UK) was used to assess the superficial blood flow (SBF) immediately before and after the process of determining CPT (Fig 1B). The circular pin electrode was removed after the process of determining CPT. SBF was measured in a round skin area of about 630 mm^2^ (diameter:15 mm) which is concentric to the circular pin electrodes. The average blood flow influx was considered to be the value of SBF. SBF increment (%) was calculated as: (SBFpost—SBF_pre_)/ SBF_pre_.

### Skin temperature

Infrared thermography (Thermovision A40; FLIR; Danderyd, Sweden) was used to assess the skin temperature (ST) before and after the process of determining CPT. ST was measured immediately after measuring SBF (Fig 1B). The ST was measured in a round area of about 630 mm^2^ (diameter:15 mm) which was the same with the area of SBF measurement. The average temperature was considered to be value of ST. ST increment (%) was calculated as: (STpost—ST_pre_)/ ST_pre_.

### Statistical analysis

The reliability analysis was performed for the measurements of CPT, SBF and ST between two experimental sessions. The relative reliability was assessed using intra-class correlation coefficient (ICC) whereas the absolute reliability was assessed using coefficient of variation (CV) and Bland-Altman analysis. These three methods are most commonly used to report reliability in test-retest studies up to date [23, 24]. The ICC two-way mixed model (type: consistency) was used to estimate the relative reliability both within and between sessions. Coefficient of variation (CV) was used for estimating the absolute reliability and is the standard deviation (SD) divided by the mean (*μ*) of each measurement for each individual. Bland-Altman analysis is an evaluation method for analyzing the limits of agreement of measurements in two experimental sessions. The limits of agreement provide an estimation of the sample error.

One-way repeated measure analysis of variance (RM-ANOVA) was used to determine differences between values of CPT in two sessions. Two-way repeated measure analysis of variance (Time and sessions were two within-subjects factors) (RM-ANOVA; SPSS v. 21.0) was used to determine differences between values of SBF and ST in two sessions. Greenhouse-Geissers method was used for the correction of non-sphericity. Bonferroni adjustment was used for multiple comparisons. Unpaired t test was used for compared the differences for CPT values between male and female subjects. The correlation between each two of CPT, SBF increment and ST increment was analyzed by Pearson correlation test. The data is presented as mean values±SD (standard deviation). P-value<0.05 was considered to be statistically significant.

## Results

**Fig 2:**
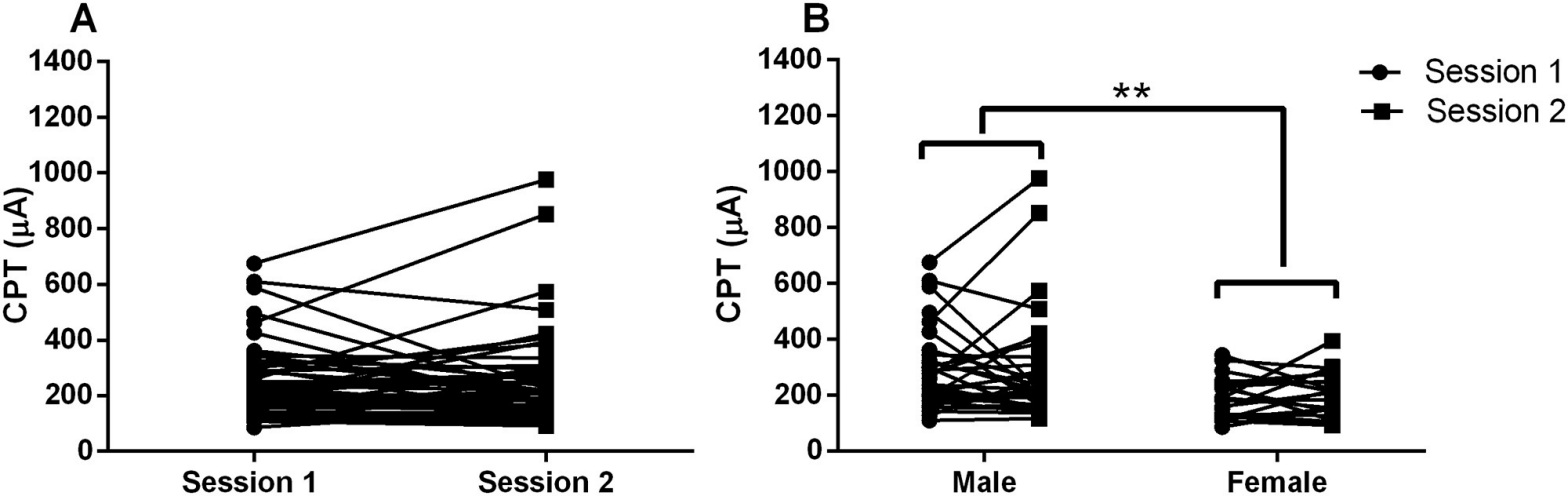
A. Current perception threshold in two sessions. B. Comparison of CPT values between males and females. **: p<0.01.

All participants completed the experiments. No side effects and discomfort were induced by the circular pin electrodes after transcutaneous electrical stimulation in all subjects. The time to finish the whole process finding the CPT value was around 15–20 minutes. The CPT values were 246.19±127.85 μA in session one and 249.57±165.02 μA in session two (mean±SD), respectively. However, no significant differences were found between two sessions (Fig 2A). CPT values of males (279.46±170.09 μA) were found to be higher than females (196.76±76.12 μA) (p = 0.002) (Fig 2B).

**Fig 3:**
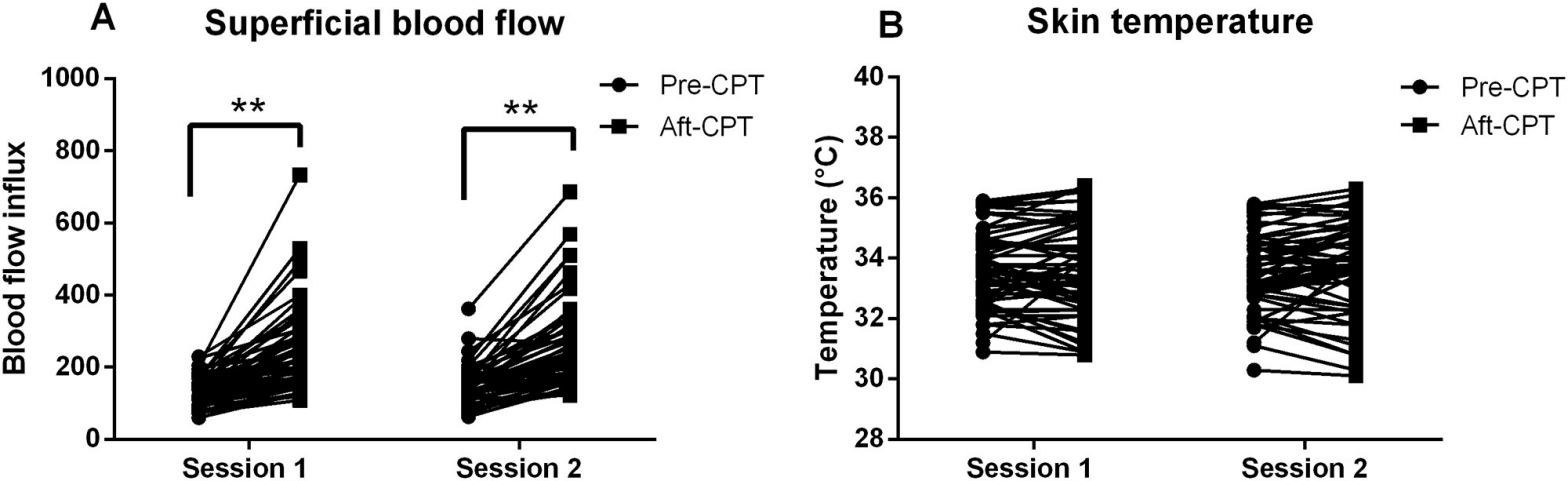
A: Superficial blood flow influx in two sessions. B. Skin temperature in two sessions. **: p<0.01.

SBF influx values were not significantly different between two sessions (session effect, F = 3.29, p = 0.075). SBF influx values were found to increase after the process of finding CPT values (time effect, F = 71.65, p<0.001). The average SBF influx value before and after the process of finding CPT values in session 1 were 138.34±40.71 and 241.09±118.49, respectively. The increment was 74.25%. The average SBF influx value before and after the process of finding CPT values in session 2 were 146.04±52.64 and 253.13±121.7, respectively. The increment was 73.32%. No interaction effect was found between session and time factors (F = 0.216, p = 0.644) (Fig 3A).

ST values were not significantly different between two sessions (session effect, F = 0.035, p = 0.852). The average ST before and after the process of finding CPT values in session 1 were 33.574±1.27°C and 33.578±1.58°C, respectively. The increment was 0.01%. The average ST before and after the process of finding CPT values in session 2 were 33.522±1.29°C and 33.684±1.54°C, respectively. The increment was 0.48%. However, no significant difference was found before and after the process of finding CPT values (time effect, F = 0.571, p = 0.453). No interaction effect was found between session and time factors (F = 3.146, p = 0.082) (Fig 3B).

**Fig 4:**
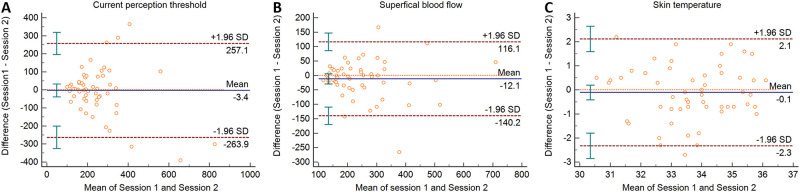
Bland-Altman plots show the absolute test-retest reproducibility of current perception threshold (A), superficial blood flow influx (B) and skin temperature (C) in two sessions.

The ICC and CV values CPT, SBF and ST are presented in Table 1. The ICC values for SBF and ST were 0.852 and 0.728, which showed good reliability. The ICC value for CPT was 0.595, which showed fair reliability. The Bland-Altman plots for CPT, SBF and ST are presented in Fig 4. According to the Bland-Altman plots, the data of CPT, SBF and ST are homoscedastic because the level of equality is within the CI of mean difference for each measurement.

**Table 1:** Reliability and correlation analysis.

Assessment measure	Correlation	CV(%)	ICC(95% confidence interval)
		Between session	Between session
CPT	CPT vs. SBF_i_ (r = -0.105, p = 0.277)	25.53	0.595 (0.393–0.742)
SBF increment	SBF_i_ vs. ST_i_ (r = 0.435, p<0.01)	12.59	0.852 (0.759–0.911)
ST increment	ST_i_ vs. CPT (r = 0.139, p = 0.149)	1.94	0.728 (0.575–0.832)

CPT: Current perception threshold; SBF: Superficial blood flow; ST: Skin temperature; SBF_i_: SBF increment; ST_i_: SBF increment; ICC: intra-class correlation coefficient; CV: coefficient of variation.

SBF increment was found to be positively correlated with ST increment (r = 0.435, p<0.01). However, CPT was not found to be correlated with ST increment (r = 0.139, p = 0.149); CPT was not found to be correlated with SBF increment (r = -0.105, p = 0.277) (Table 1).

## Discussion

We explored the reliability of current perception threshold (CPT) measurements using the circular pin electrodes with at least one week interval between sessions and measured the superficial blood flow and skin temperature changes as secondary neurogenic inflammation outcomes caused by CPT measurements in the present study. We found that the reliability of CPT measurements using circular pin electrodes was fair. Superficial blood flow was significantly increased after the process of CPT measurement using low intensity electrical stimulation. CPT values of males were found to be higher than females.

CPT testing is a type of quantitative sensory testing (QST) technology, and its clinical application has two major applications: one is metabolic peripheral neuropathy, and the other is chronic embedded pressure induced peripheral neuropathy, such as diabetic peripheral neuropathy and carpal tunnel syndrome [25, 26]. CPT values have been reported to have gender differences. In our study, we found that CPT values of males were higher than females. This is in line with other study that men and women differ in their responses to pain with increased pain sensitivity and many more painful diseases commonly reported among women [27]. A previous study using Neurometer^®^ device with large surface electrode measured at the left index finger also reported that women showed lower CPT values than men at 250 Hz and 5 Hz frequency stimulus which are correlated to small fiber function [1, 18]. However, another study showed that no statistical gender differences were shown for the median nerve [28]. Other studies using thermal perception threshold also showed that men had a higher perception threshold compared to women [29, 30]. The possible reasons could include: First, like all subjective sensory tests, it has variability. Second, it was reported that the density of epidermal small nerve fibers is lower in men compared with women which may explain that the higher CPT values existed in men compared with women [31]. Third, different studies measured different position of the body, and the densities of nerve fibers were different in different parts of human body which may lead to the variabilities of sensation and disagreement between studies [32].

Superficial blood flow (SBF) and skin temperature (ST) were measured manifesting the neurogenic inflammation response. SBF was found to be significantly increased after the process of finding CPTs in the present study. This is in agreement with previous studies that low intensity nonpainful electrical stimulation could increase cutaneous blood flow [12, 33]. Electrical stimulation may activate peptidergic nerve fibers which can release inflammatory mediators, e.g., calcitonin gene-related peptide (CGRP) or substance P (SP) [11]. SP can cause vasodilatation and increase in vascular permeability [34]. CGRP has been reported to potentiate vascular permeability caused by other mediators [35]. These vascular effects of SP and CGRP taken with their localization in peptidergic afferents has led to their implication in increasing SBF caused by electrical stimulation. Previous studies have shown that the activation of mechano-insensitive C-nociceptors (“silent” nociceptors) can cause axon reflex flare by high intensity painful electrical stimulation [36–38]. It has been shown that the Aδ-fibers could be activated by low intensity electrical stimulation (i.e., twice perception threshold) [11]. In our study, the process of finding CPTs involved a repeated electrical stimulation with increasing and decreasing intensities surrounding the perception threshold. The maximum stimulation intensities could reach 10% above the perception threshold. Using the pin electrodes, the most probable fibers to be activated are A-δ fibers [38]. Likewise, thermal perception threshold testing has been used for A-δ fibers (skin cooling, cold detection threshold) and C fibers (skin warming, warm detection threshold) assessments [39]. Previous studies also showed that C-fibers could be activated by mall area electrodes and triangular stimulation pulses [40, 41]. Therefore, the coactivation of C-fibers cannot be excluded. Therefore, a certain amount of both peptidergic C- and A-δ fibers might be involved which led to the increase of SBF in the present study [42]. ST was also found to be increased after the process of determining CPTs, however, no significant differences were found. Previous studies also showed that nonpainful electrical stimulation could not affect skin temperature [12, 33]. This implicates that ST might not a sensitive parameter showing the neurogenic inflammation response caused by electrical stimulation or [21]. Alternatively, ST could be easily affected by ambient temperature which showed that the importance of controlling ambient temperature to ensure reliable and accurate results [43]. According to ICC values, due to the fact that stimulated skin area might not be exactly the same between two sessions, different amount of small nerve fibers might be activated. This may lead to a slightly different extent of SBF and ST between sessions.

SBF increment was found to be positively correlated with ST increment in the present study. Therefore, the increase of SBF was accompanied with a mild increase of ST after the process of finding CPTs. However, CPT was not found to be correlated with SBF and ST. Therefore, the electrical stimulation at low intensities, i.e., surrounding the perception threshold, might not affect the amount of activated peptidergic afferents or the release of SP/CGRP when CPTs increased or decreased for the same subject. In other words, a higher CPT might not induce a higher SBF or ST in the present study.

The reliability analysis showed that the ICC values of SBF and ST measurements were high with good reliability. However, the ICC value of CPT measurement between sessions was lower than ICC values of SBF and ST measurements. The CV value of CPT was higher than that of SBF and ST. It was reported that reproducibility variations in pain detection could be related in the way people produce decision making on what a painful stimulus is [44]. Therefore, the larger variations of CPT measurements could be due to the protruding pins induced a mild pinprick sensation which may interfere subjects’ judgments to CPTs. Furthermore, pain perception is affected by many different factors which may cause the diurnal variations [45, 46]. These factors include vegetative, hormonal variables as well as psychological state factors which may cause pain perception differences in two different days [45, 46]. We tried to place the circular in electrodes in the same skin location in two sessions, however, even the very small shift of the location could lead to a different impedance which could influence the current stimulation intensity. Therefore, a special caution should be taken that the stimulated skin area should be better consistent in the longitudinal studies when measuring CPT using circular pin electrodes. SBF and ST showed higher ICC values and lower CV values in our study. This indicated that SBF and ST might be less affected by the skin location if the intensity of electrical stimulation reached perception threshold. According to the results of repeated measures ANOVA, however, no significant differences were found for CPT, SBF and ST between sessions. The Bland-Altman plot also showed that the level of equality was within the CI indicating the agreement between two sessions for CPT, SBF and ST measurements.

There are still several limitations in the present study. Firstly, a control circular pin electrode without electrical stimulation was not set on the contralateral arm. The pins of the electrode also produced pinprick sensation which might also irritate the skin. Therefore, a control side without electrical stimulation would help to eliminate the superficial blood changes that might be caused by the pinprick stimulation from the pins. Secondly, it is a pity that the circular pin electrodes need to be removed after finishing session one and tried to replace on the same place in session two. This might lead to a slight place shift which could not avoided completely. Maybe we would not need to move the electrodes if the test-retest study was performed within several hours. However, from a clinical part of view, performing re-test in two different days may have more clinical significance than performing the re-test within several hours in longitudinal observation. However, the consistence of measurements between sessions, e.g., the same measured skin area and time of the day, should be kept during the observation period. Lastly, further studies comparing the assessing function between circular pin electrodes and conventional Neurometer method should be conducted to confirmed its priority in assessing the function of thin nerve fibers.

## Conclusions

The measurement of current perception threshold (CPT) using circular pin electrodes was conducted in our study to assess human sensory function, especially used to assess the function of cutaneous small nerve fibers. The reliability of CPT measurement using circular pin electrodes is fair, and need consistence of measurements in longitudinal studies when assessing small fibers mediated sensory changes.
